# Fracture of Coracoid Process of Scapula

**Published:** 1886-05

**Authors:** L. E. Borcheim

**Affiliations:** Atlanta, Ga.


### FRACTURE OF CORACOID PROCESS OF SCAPULA.

By L. E. BORCHEIM, M. D., ATLANTA, GA.

Instances of fracture of this process of bone are so rare that if, from no other motive, their scarcity entitles them to publication when they do occur.

Packard, of Philadelphia, mentions (Vol. IV. Internat. Encycl. Surg., p. 114) that he saw three cases in 316 fractures, and Erichsen (Surgery, Vol. I, p. 410) says that there are but ten or twelve unequivocal cases on record. As is usually the case, this accident occurred as the result of direct violence as follows:

S. D., aged 66 years, while walking along the street, was struck full on the right shoulder by a runaway horse, lifting him bodily from off his feet and violently throwing him against an iron column; upon examination, found, among other injuries of which I shall make no mention, pain about the shoulder very severe, patient complaining that his arm was broken, but found that no false point of motion was apparent until I came to the coracoid process, manipulation about which causing exquisite pain. Some crepetation was made out, and a slight degree of displacement. Considering the strength of the muscles attached to this process of bone, we would naturally expect to find great displacement, but this was not the case, and is explained by the fact that it gives insection to ligaments whose fibres are expanded over it, thus maintaining in a measure its integrity. (Erichsen.)

The treatment consisted in relaxing the peptoralis minor, eoraco brachilis and bicips, which was accomplished by placing the arm in a large triangular sling, flexing the forearm on the arm and keeping it close to the chest by a turn of roller bandage.

Dr. Gaston saw the case with me, and concurred with my diagnosis.

